# Prevalence of Electronic Cigarette Smoking Among Students of Shaqra University, Saudi Arabia

**DOI:** 10.7759/cureus.58996

**Published:** 2024-04-25

**Authors:** Fahad A Algassim, Mohammed E Alhowaiti, Adel S Alotaibi, Ibrahim M Alanazi, Abdulmajeed A Almutairi, Abdulaziz A Alanezi, Abdulmajeed M Almansour, Muath A Alammar

**Affiliations:** 1 Family Medicine, Shaqra University College of Medicine, Shaqra, SAU

**Keywords:** dawadmi, shaqra, prevalence, smoking, e-cigarettes

## Abstract

Background

Electronic cigarettes are devices that use a flavored nicotine solution instead of burning tobacco leaves. Since their emergence, e-cigarettes have gained popularity in Saudi Arabia, particularly among young adults. Recently, many non-smoking youths have begun to use e-cigarettes as an alternative social behavior. Recent studies have confirmed that e-cigarettes have harmful effects on the respiratory system. Approximately 48.5 million Europeans have used an e-cigarette at least once while 7.5 million Europeans currently use e-cigarettes. This study aims to assess the prevalence of e-cigarette use and possible addictiveness among Shaqra University students.

Methodology

This is a cross-sectional study conducted at Shaqra University in 2021. A total of 290 students (18 years old and older) from Shaqra University were included in our study. The subjects were selected through simple random sampling. A self-administered online questionnaire related to e-cigarettes was used.

Results

Completed questionnaires were obtained from 290 students (average age of 20.2 ± 1.8 years). A total of 58 (20.1%) of the respondents were e-cigarette users. The obtained results showed that the mean age of e-cigarette smokers was 20.5 years, e-cigarette usage significantly differed between age groups (*p < 0.001*), and the highest prevalence of e-cigarette smoking was in Shaqra Governorate (i.e., 13.1%).

Conclusion

It's vital to acknowledge that the capacity for addiction to e-cigarettes is comparable to traditional smoking and other nicotine-containing items. It's essential to consistently observe students and smokers to better understand the effects of vaping patterns on this specific group

## Introduction

The e-cigarette is a device consisting of a mouthpiece, an atomizer, a cartridge, and a battery, which emits nicotine in the form of an aerosol. The cartridge contains a liquid solution containing nicotine, flavorings, and other chemicals. When heated by the atomizer, the liquid transforms into an aerosol that users inhale [1,2]. Adolescents' use of electronic cigarettes (e-cigarettes) has risen, likely due to the decline in traditional smoking over the past decade [3,4].

Research indicates that non-smokers using e-cigarettes may inadvertently promote the uptake of other tobacco products, which can have adverse health effects and lead to conditions like lung cancer. A study conducted in the United States found that 21.8% (178,850 out of 820,414) had initiated cigarette smoking within two years of using e-cigarettes. The majority of cases of lung diseases worldwide, including in Saudi Arabia, are linked to tobacco use [4-9].

In addition, respiratory consequences encompass conditions such as status asthmaticus and pneumothoraces; however, the most frequently documented and severe adverse effect is diffuse parenchymal lung disease [10].

During the summer of 2019, the EVALI (e-cigarette or vaping use-associated lung injury) outbreak caused 2807 instances of unexplained sudden lung injury primarily in young, healthy individuals, leading to 68 fatalities. Epidemiological investigation into the outbreak revealed a correlation with vaping [11].

Due to limited data on the prevalence of e-cigarette use among Shaqra University students and Saudi Arabian students, and their harmful impact on health, there is an urgent need for a new study to bridge the knowledge gap regarding e-cigarette usage among Shaqra University students, and how they perceive it in terms of safety.

General objectives

The objectives of this study are: 1) to assess the prevalence of e-cigarette use among students at Shaqra University in Shaqra, KSA, 2) to evaluate the factors contributing to the increase in e-cigarette usage, and 3) to elucidate how e-cigarettes are perceived in terms of safety and addictiveness.

## Materials and methods

This study is a cross-sectional study conducted among Shaqra University students in Shaqra Province of Saudi Arabia. It assesses the prevalence of e-cigarette use and factors contributing to increased usage in 2021. We selected the subjects by a random sampling method of Shaqra University students where each student had to fill out a survey. The survey measured the objective as follows: Prevalence of e-cigarette use among Shaqra University students: Questions 5, 6, 7, 8, 10, 16, 17, and 19 help gauge the prevalence of e-cigarette usage among students. Factors contributing to the increase in e-cigarette usage: Questions 9, 10, 11, 12, 13, 14, 18, and 19 explore various factors such as awareness, social influence, perceptions of safety, and attractiveness of e-cigarettes. How e-cigarettes are perceived in terms of safety and addictiveness: Questions 8, 11, 12, 13, 14, and 15 delve into perceptions regarding the safety of e-cigarettes compared to traditional cigarettes, and their role in smoking cessation, initiation, and continuation. We used the total count method to include all adult males and females who agreed to answer the questionnaire in this study, which was 25%. The sample size was determined based on the anticipated prevalence of e-cigarette use among Shaqra University students in Saudi Arabia, a confidence interval of 95%, an error not exceeding 5%, and an expected nonresponse rate of 50%. We used a coefficient chi-squared test to determine factors that would contribute to increasing the usage of e-cigarettes and to evaluate the perception of e-cigarette safety.

The basis of this questionnaire is multifaceted, focusing on several key aspects related to e-cigarette usage among Shaqra University students. Questions 1, 2, and 3 gather basic demographic data such as sex, age, and governorate. Questions 5, 6, and 7 assess behavioral habits like current usage of e-cigarettes, regular smoking, and shisha. Questions 8 and 9 explore awareness and sources of information about electronic cigarettes. Questions 10 and 18 delve into the influence of friends and relatives who use e-cigarettes and factors attracting users to them. Questions 11 through 15 investigate perceptions regarding the role of e-cigarettes in smoking cessation, initiation, continuation, safety compared to regular cigarettes, and opinions on regulation. Question 19 examines whether recommendations from e-cigarette users influence others to purchase them.

Statistical analysis

We performed data collection, organization, and analysis using a Microsoft Excel program (version 16.0.16327.20200; Microsoft Corporation, Redmond, WA, US). We conducted a statistical analysis using IBM SPSS (version 28.0.1.1 Armonk, NY: IBM Corp). Basic demographics, smoking, and e-cigarette and other tobacco product use were summarized using descriptive statistics. The coefficient and chi-squared tests were used for categorical variables. The criterion for significance was set at p<0.05.

Ethics approval and consent

This study was in accordance with the ethical standards of the authors’ institutional research committee. Informed consent was obtained from all the participants. This study was approved by the scientific research committee at Shaqra University (NO: ERC_SU_20220096).

## Results

We obtained completed questionnaires from 290 students from Shaqra University with an 89.78% response rate. Their average age was 20.20 ± 1.77 years (range of 18-25 years of age); 192 (66.2%) of them were males and 98 (33.8%) were females. Table 1 shows the distribution of age, gender, and governorate.

**Table 1:** Demographical distribution This table shows the mean age and sex distribution by percentage and number of participants in each Governorate. N = number

Governorates	Mean age (SD)	Male N (%)	Female N (%)
Afif	19.75 (1.38)	5 (1.7)	3 (1)
Al-Muzahimiyah	21.50 (2.1)	0	2 (0.6)
Al-Quwaiiyah	19.26 (1.6)	10 (3.4)	9 (3.1)
Dawadmi	19.85 (1.5)	65 (22.4)	51 (17.6)
Dhurma	20.75 (2.9)	1 (0.3)	3 (1)
Huraymila	19.83 (1.6)	3 (1)	9 (3.1)
Sajir	20.50 (1.4)	7 (2.4)	3 (1)
Shaqra	20.70 (1.9)	101 (34.8)	17 (5.8)
Thadiq	21.00 (0)	0	1 (0.3)

The results show one-fifth of the respondents (58) had used e-cigarettes. The highest prevalence of e-cigarette use is in the Shaqra Governorate 13.1% (38), followed by Dawadmi 5.1% (15), which are Governorates that have the largest branches of Shaqra University and have the largest number of students, some of whom are from other cities. Meanwhile, the lowest prevalence (0) was in Al-Muzahimiyah, Dhurma, Sajir, and Thadiq. A total of 51 (17.5%) of the participants reported using traditional cigarettes and the use of shisha was reported by 13.1% (38) of the participants. Table 2 shows the prevalence of e-cigarette and other tobacco product use.

**Table 2:** E-cigarette user distribution This table shows the number and percentage of e-cigarette user distribution based on sex and Governorates, with the mean age of the participants. N = number

Sociodemographic characteristic		Are you a current user of electronic cigarettes? N (%)	
		Yes	No
Mean Age (SD)		20.53 (1.90)	20.13 (1.73)
Sex	Male	56 (19.3)	136 (46.9)
	Female	2 (0.7)	96(33.1)
Governorate	Afif	1 (0.3)	7 (2.4)
	Al-Muzahimiyah	0	2(0.7)
	Al-Quwaiiyah	2 (0.7)	17 (5.9)
	Dawadmi	15 (5.2)	101 (34.8)
	Dhurma	0	4 (1.4)
	Huraymila	2 (0.7)	10 (3.4)
	Sajir	0	10 (3.4)
	Shaqra	38 (13.1)	80 (27.6)
	Thadiq	0	1 (0.3)

We used a coefficient test to assess the factors that would contribute to increasing the usage of e-cigarettes. E-cigarette smoking was the dependent variable while we used age, gender, recommendation for e-cigarettes, and smoking habits as the predictors. The obtained results show that the mean age of e-cigarette smokers is 20.5 (p < 0.001), and those who use regular cigarettes tend to also use e-cigarettes as compared to those who use other types of tobacco products (p < 0.001). Most e-cigarette users (34/58; 58%) state that they are attracted to them because “they have no smell” (p < 0.001), and some of them (17/58; 29.3%) say that more than 60% (p < 0.001) of their friends and relatives use e-cigarettes. These data are presented in Tables 2, 3.

**Table 3:** Correlation of e-cigarette usage This table shows the number and percentage of e-cigarette users in correlation with multiple variables using the coefficient test with* P<*0.05 set as significant. N = number

Characteristic		Are you a current e-cigarette user? N (%)		p-value
		Yes	No	
Regular cigarette smokers	No	25 (8.6)	214 (73.8)	< .001
	Yes	33 (11.4)	18 (6.2)	
Shisha smokers	No	29 (10)	223 (80.3)	.130
	Yes	29 (10)	9 (3.1)	
Other tobacco product user	No	37 (12.8)	213 (73.4)	.746
	Yes	21 (7.2)	19 (6.6)	
Percentage of friends and relatives using e-cigarettes	0%	1 (0.3)	62 (21.40)	.005
	<20%	12 (4.1)	105 (36.2)	
	20-40%	14 (4.8)	46 (15.9)	
	40-60%	14 (4.8)	11 (3.8)	
	>60%	17 (5.9)	8 (2.8)	
What attracts you to e-cigarettes?	Not using it	0	221 (76.2)	< .001
	Flavor	16 (5.5)	5 (1.7)	
	Price	8 (2.8)	3 (1)	
	Have no smell	34 (11.7)	3 (1)	
E-cigarette recommendation	No	28 (9.7)	169 (58.3)	< .001
	Yes	30 (10.3)	63 (21.7)	
Sex	Male	56 (19.3)	136 (46.9)	.902
	Female	2 (0.7)	96 (33.1)	
Mean age (SD)		20.53 (1.90)	20.13 (1.73)	< .001

A chi-squared test was used to assess the perception of e-cigarette safety. The findings reveal that 102 participants (35.2%) believe e-cigarettes aid in smoking cessation (p < 0.001) while 193 participants (66.6%) express concerns that e-cigarettes may encourage smokers to continue, potentially hindering quitting efforts (p < 0.001) while 230 (79.3%) participants think that e-cigarettes encourage smoking initiation by those who have never smoked (P < 0.016). A total of 213 (73.4%) participants think that e-cigarettes should be regulated in public areas (p < 0.001). These data are presented in Table 4.

**Table 4:** Perception of e-cigarette safety This table shows the perception of e-cigarette safety and addictiveness among the participants represented by numbers and percentages using the chi-squared test with *P<*0.05 set as significant. N = number

Characteristic		Are e-cigarettes safer than regular cigarettes? N (%)		p-value
		Yes	No	
Are e-cigarettes a helpful aid for smoking cessation?	No	33 (11.4)	155 (53.4)	< .001
	Yes	66 (22.8)	36 (12.4)	
Are e-cigarettes encouraging smoking continuation among smokers who might have quit otherwise?	No	40 (13.8)	57 (19.7)	< .001
	Yes	59 (20.3)	134 (46.2)	
Are e-cigarettes encouraging smoking initiation in those who have never smoked?	No	29 (10)	31 (10.7)	.016
	Yes	70 (24.1)	160 (55.2)	
Should e-cigarettes be regulated in public areas?	No	51 (17.6)	26 (8.9)	< .001
	Yes	48 (16.6)	165 (56.9)	

## Discussion

E-cigarette use is currently increasing among young adults in Saudi Arabia because of the spread of the misconception that e-cigarettes are safer compared to regular tobacco cigarettes [12]. Recent reports have shown that e-cigarettes have similar hazards to tobacco smoking [13]. Thus, it is important to understand the features of e-cigarettes in Saudi Arabia.

In this study, we assessed the prevalence of e-cigarette smoking among Shaqra University students in 2021. The prevalence of e-cigarette use was 20.1% (58) among students from different Shaqra University branches. This percentage is comparable to the previously reported 27.7% (279/1007) in health science colleges in Jeddah [2,4]. Another study was performed at Alfaisal University in Riyadh, whose authors reported a 12.2% (49/401) rate of e-cigarette use [14]. In the study on health professional students in the United States, approximately 24.2% (206/853) of the participants confirmed that they had tried e-cigarettes [4,15]. Similarly, in a study performed in the United Kingdom, the authors reported that 21.5% (884/4117) of the participants had used e-cigarettes [16]. A total of 10.6% (27/256) of the participants stated that they had tried e-cigarettes at Qassim University in Saudi Arabia [17], and 17.8% (51) of the participants smoked regular cigarettes. The percentage of regular cigarette users decreased because there is a policy banning smoking at all facilities on a college campus. Therefore, it is predicted that e-cigarettes have gained more popularity than regular cigarettes due to flavors that give them a pleasant taste, lack of smell, and absence of residue.

In this study, 35.6% (103) of the users considered e-cigarettes to be a helpful tool for stopping smoking entirely. In the city of Jeddah, which is in the western region of Saudi Arabia, 42.7% (119/279) of students used e-cigarettes to quit smoking [2]. In addition, 11% (28/256) of students in a study at Qassim University recommended e-cigarettes as a better alternative to tobacco [4]. Twenty-three point one percent (23.1%; 197/853) of American university students and approximately 50.7% (~800,000) of smokers in Canada also used e-cigarettes for this reason [2,15,17,18]. Similarly, authors of a study performed in Poland reported that 58.7% (56/95) of students used e-cigarettes as a smoking cessation method [19]. These results suggest that students need more education about e-cigarettes because these devices are not approved by the U.S. Food and Drug Administration as a tool to stop smoking [14].

The obtained data show that 79.5% (230) of students believe that e-cigarettes encourage starting smoking for those who have never smoked at all. Similarly, other studies confirm that there is an association between e-cigarettes and the initiation of smoking [6]. This study also shows that e-cigarettes are possibly addictive and may encourage smoking continuation among smokers who may have already quit smoking. When questioning the students on their awareness of the addictiveness of e-cigarettes, 48.9% (418/854) of them are aware that both regular cigarettes and e-cigarettes are equally addictive [2] because e-cigarettes are devices that contain a nicotine solution [20]. Nicotine acts on a nicotinic cholinergic receptor in the brain and triggers the release of neurotransmitters (e.g., dopamine, GABA, and glutamate), which are important in the development of nicotine dependence [21].

A cross-sectional, survey-based study has been previously performed in the United States on 658 medical students from the University of Minnesota to evaluate student knowledge about e-cigarettes. A total of 97 participants (14.7%) stated that they had used e-cigarettes in the past, and four participants (0.6%) confirmed that they were current users. Significantly, most of the participants (619; 95%) felt that they did not receive adequate education about e-cigarettes in medical school. A total of 185 participants (28.2%) stated that e-cigarettes were helpful in smoking cessation, and 258 participants (39.3%) believed that e-cigarettes had lower cancer risks than traditional cigarettes. This study suggests that there is a lack of knowledge about e-cigarettes among medical students at the University of Minnesota and indicates the need to include the subject of e-cigarettes in the medical school curriculum [4,22].

In this study, we found that 34.9% (101) of the students believed that e-cigarettes are safer than regular cigarettes. According to a study, 10% (1015/10146) of Mexican middle school students have tried e-cigarettes and 19% (1928) think they are less harmful than traditional smoking [2,23]. In a general population study in the United Kingdom, 67% (572/854) of the respondents had the same opinion, believing that e-cigarettes were less dangerous than traditional cigarettes [2,15]. Therefore, additional studies are needed to determine whether e-cigarettes are safer than regular cigarettes or whether both are equally hazardous.

Limitation 

We recognize that our study is limited to a particular group with a high level of education within a specific geographic location, thus reducing the applicability of our findings to the broader population of Saudi Arabia. Additionally, our reliance on self-reported data introduces potential biases such as recall and social desirability. Therefore, conducting a larger study encompassing the diverse population of Saudi Arabia is necessary for more comprehensive insights.

## Conclusions

E-cigarette usage surpasses traditional smoking (20.1% versus 17.8%) among Shaqra University students, indicating a growing trend. The allure of e-cigarettes lies in their odorless nature and diverse range of flavors. Interestingly, some students turn to e-cigarettes as a means to quit smoking. Nonetheless, it's crucial to recognize that e-cigarettes carry a similar addictive potential to conventional smoking and other nicotine-based products. It's imperative to continually monitor students and smokers to gain deeper insights into the impact of vaping trends on this particular demographic.

## Appendices

Questionnaire

1.      What is your gender?

a.       Female

b.      Male

2.      What is your age?

a.       18

b.      19

c.       20

d.      21

e.       22

f.        23

g.      More than 24

3.      Governorate:

a.       Shaqra

b.      Dawadmi

c.       Afif

d.      Sajir

e.       Al Quwaiiyah

f.        Thadq

g.      Huraymila

h.      Dhurma

4.      College:

a.       College of Medicine

b.      College of Pharmacy

c.       College of Engineering

d.      College of Science and Arts

e.       College of Computing and Information Technology

f.        College of Applied Medical Sciences

g.      Deanship of Preparatory Year

h.      College of Science and Humanities

i.        College of Business Administration

5.      Are you a current user of electronic cigarettes?

                                           Yes                          No

6.      Are you a current smoker “regular cigarette“?

                                           Yes                          No

7.      Are you a current Shisha smoker?

                                           Yes                          No

8.      Do you know what is the electronic cigarette?

                                           Yes                          No

9.      How did you first learn about electronic cigarettes?

a.       Friends

b.      Advertising

c.       Family

d.      Other

10.  About what percentage of your friends or relatives use electronic cigarettes?

a.       No one: 0%

b.      Less than 20%    

c.       20% to 40%

d.      40% to 60%

e.       More than 60%

11.  Do you think electronic cigarettes are a helpful aid for smoking cessation?

                                           Yes                          No

12.  Do you think electronic cigarettes are encouraging smoking initiation in those who have never smoked?

                                           Yes                          No

13.  Do you think electronic cigarettes are encouraging smoking continuation among smokers who might have quit otherwise?

                                           Yes                          No

14.  Do you think electronic cigarettes are safer to use than regular cigarettes?

                                           Yes                          No

15.  Do you think that electronic cigarettes should be regulated in public areas?

                                           Yes                          No

16.  Have you ever used other tobacco products?

                                           Yes                          No

17.  Have you ever used electronic cigarettes in places where smoking is prohibited?

                                           Yes                          No

18.   If you are an electronic cigarette user, what attracted you to it?

a.      It has no smell

b.      Prices

c.      Flavors

d.     I am not using it

19. Does someone who uses electronic cigarettes recommend you to buy one?

                                           Yes                          No
